# A Functional Single Nucleotide Polymorphism in the 3′ Untranslated Region of the Porcine JARID2 Gene Is Associated with Aggressive Behavior of Weaned Pigs after Mixing

**DOI:** 10.3390/ijms25010027

**Published:** 2023-12-19

**Authors:** Huan Yang, Chunlei Zhang, Xiaohuan Chao, Jing Zhao, Mingzheng Liu, Jiahao Chen, Shuhan Liu, Tianshuo Wang, Asim Muhammad, Allan P. Schinckel, Bo Zhou

**Affiliations:** 1 College of Animal Science and Technology, Nanjing Agricultural University, Nanjing 210095, China; 2021105038@stu.njau.edu.cn (H.Y.); 2020105039@stu.njau.edu.cn (C.Z.); 2021205020@stu.njau.edu.cn (X.C.); 2019105039@stu.njau.edu.cn (J.Z.); 2020205018@stu.njau.edu.cn (M.L.); 2021105039@stu.njau.edu.cn (J.C.); 2022105002@stu.njau.edu.cn (S.L.); 2022805106@stu.njau.edu.cn (T.W.); 2022105147@stu.njau.edu.cn (A.M.); 2 Department of Animal Sciences, Purdue University, West Lafayette, IN 47907, USA; aschinck@purdue.edu

**Keywords:** pig, aggressive behavior, JARID2, SNP, miR-9828-3p

## Abstract

In pig production, pigs often show more aggressive behavior after mixing, which adversely affects animal welfare and growth performance. The Jumonji and structural domain-rich AT interaction domain 2 (JARID2) gene plays an important role in neurodevelopment in mice and various psychiatric disorders in humans. The JARID2 gene may impact the aggressive behavior of pigs. By observing the behavior of 500 weaned pigs during the first 72 h after mixing, the ear tissue samples of the 12 most aggressive and 12 least aggressive pigs were selected for DNA resequencing based on the intensity of their aggressive behavior. Large group correlation analysis indicated that the rs3262221458 site located in the 3′-UTR region of the porcine JARID2 gene has a strong relationship with the aggressive behavior of weaned pigs. Pigs with the mutant TT genotype of rs3262221458 have more aggressive behavior than those pigs with the GG and GT genotypes. The dual luciferase assay indicated that the luciferase activity of the plasmids containing the G allele of rs326221458 was significantly less than that of plasmids containing the T allele of rs326221458 and control groups. The binding ability of miR-9828-3p to sequences containing the T allele was less than that of sequences containing the G allele. The overexpression of miR-9828-3p in porcine neuroglial cells (PNGCs) and PK15 cells significantly decreased the mRNA and protein levels of the JARID2 gene. In addition, miR-9828-3p inhibited the proliferation of PNGCs. After inhibiting miR-9828-3p, the mRNA and protein expression levels of JARID2 increased, and the proliferation of PNGCs showed an opposite trend to the cells that forced the expression of miR-9828-3p. In addition, interference with the JARID2 gene by siRNA can effectively inhibit the proliferation of PNGCs. In summary, we found that the rs326221458 locus regulates the expression of the JARID2 gene by affecting the binding of miR-9828-3p and the JARID2 gene, thereby affecting the aggressive behavior of weaned pigs after mixing.

## 1. Introduction

In intensive pig farming, the animal welfare issues arising from the aggressive behavior of pigs have become increasingly prominent. Such behavior typically occurs after mixing, with the most frequent instances of aggression occurring during the first few hours after mixing [1]. Aggressive behavior in pigs commonly results in physical injuries and skin lesions, and in extreme cases, a bacterial infection of the wound could cause the death of the pigs and increase treatment costs [2,3]. Studies on pig aggressive behavior indicate that such behavior has a heritability of 0.3 to 0.4 [4,5]. Identifying molecular genetic markers related to this behavior can facilitate the genetic selection of less aggressive pigs [6].

In the screening of candidate genes for pig aggressive behavior, the porcine Jumonji and AT-rich interaction domain containing 2 (JARID2) gene has strong support as a primary candidate gene. In 1995, the JARID2 gene was discovered and named in mice with neural tube development defects [7]. The porcine JARID2 gene is located at 11,358,243–11,601,715 bp of chromosome 7 and contains a nuclear localization signaling (NLS) structural domain, strong transcriptional repressor structural domain, AT-interacting structural domain (ARID), zinc finger (C5HC2), JmjN structural domain, and JmjC structural domain [8].

During the development of the nervous system, the JARID2 gene cooperates with the Polycomb repressive Complex 2 (PRC2) complex to maintain the differentiation of progenitor cells into various types of neuronal cells, regulating the order and time pattern of the emergence of various neurons [9,10]. The JARID2 gene is mainly expressed in neurons, especially at the junction of the forebrain and hindbrain, during mouse embryonic development. Mutation of the JARID2 gene caused abnormalities in the neural groove at the midbrain–hindbrain junction in mice and increased Cyclin D1 (CCND1) protein expression [11,12]. When embryonic stem cells failed to express JARID2 normally, 58 differentially expressed genes related to early embryonic development, which were primarily associated with forebrain biogenesis and axon guidance, were detected [13]. In humans, JARID2 is in close proximity to Dysbindin (DTNBP1), which is strongly associated with schizophrenia (SCZ). There was a significant increase in the short allelic sequences with fewer than 11 repeats of the JARID2 gene in the SCZ patients. JARID2 has been considered a candidate gene for 6p22.3-linked to SCZ [14]. Additionally, the single nucleotide polymorphism (SNP) rs2237126 on the human JARID2 gene has been associated with autism spectrum disorders and clinical neurodevelopmental syndromes. People with autism are usually associated with many abnormal behaviors, including aggression [15]. Therefore, the JARID2 gene plays an important role in the development of the nervous system and the occurrence of human psychiatric disorders. Aggressive behavior is a major feature of autistic patients and is evident in more than 25% of autistic patients. This has also been confirmed in many mouse models. Judging from the existing research results, the JARID2 gene has a certain correlation with the aggressive behavior of humans, mice, and other species, but there is no report on the aggressive behavior of pigs [15]. We speculate that the JARID2 gene may also be involved in regulating aggressive behavior in pigs.

MicroRNAs (miRNAs) regulate gene expression through translational repression, or mRNA deadenylation and mRNA decay [16]. There is growing evidence that miRNAs are associated with neurodevelopment and the development of various psychiatric diseases [17,18]. Overall levels of miRNAs in the prefrontal cortex have been reported to be down-regulated in patients with major depressive disorder following suicide compared to patients who died without psychiatric illness [19].

In this study, we observed the behavior of 500 weaned pigs during the first 72 h post-mixing. We explored the association between the genotypes of an SNP in the JARID2 gene and aggressive behavior in pigs following mixing. The objective of this study was to elucidate the molecular mechanism by which the functional SNP on the JARID2 gene influences aggressive behavior in weaned pigs. To achieve this, we first identified a functional SNP in the JARID2 gene linked to aggression in weaned pigs after mixing and then investigated the molecular regulation mechanisms of this aggression.

## 2. Results

### 2.1. Association Analysis of SNPs of the JARID2 Gene with Aggressive Behavior of Pigs after Mixing

**Figure 1:**
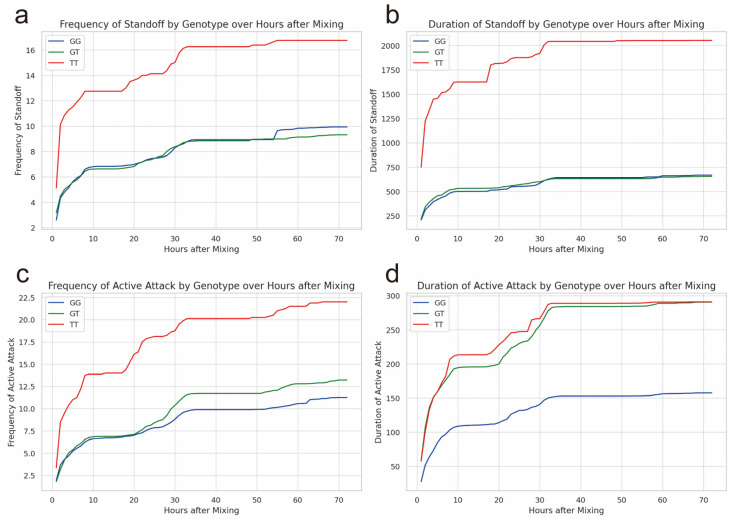
The behavioral indicators of weaned pigs with different genotypes (GG, GT, TT) of rs326221458 in the JARID2 gene during the first 72 h after mixing. (**a**) Accumulated frequency of standoff of pigs with the GG, GT, or TT genotype. (**b**) Accumulated duration of standoff (s) of pigs with the GG, GT, or TT genotype. (**c**) Accumulated frequency of active attacks of pigs with different genotypes. (**d**) Accumulated duration of active attacks (s) of pigs with different genotypes (GG, GT, TT).

**Figure A1:**
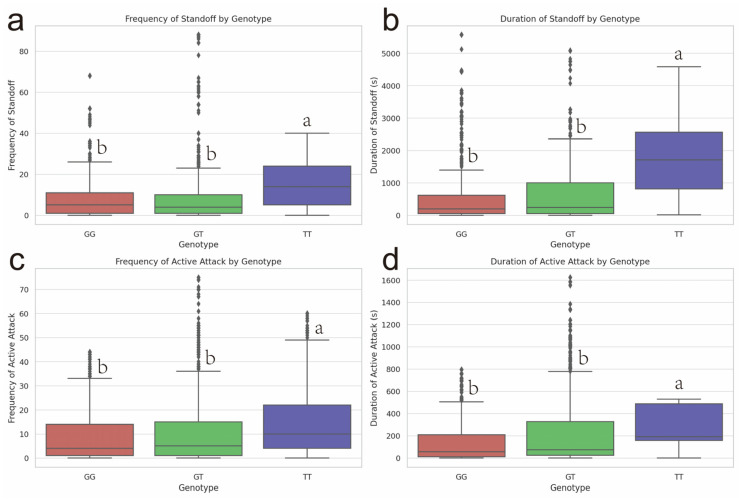
Aggressive behavior indicators between pigs with different genotypes of rs326221458. (**a**) The frequency of standoff in pigs with GG (8.18), GT (8.00), and TT (14.93) genotypes of rs326221458 (Kruskal–Wallis Test H= 258.58, *p* < 0.001). (**b**) The duration of standoff in pigs with GG (580.7 s), GT (585.8 s), and TT (1882.6 s) genotypes of rs326221458 (H = 804.18, *p* < 0.001). (**c**) The frequency of active attack in pigs with GG (8.74), GT (9.99), and TT (18.12) genotypes of rs326221458 (H = 134.01, *p* < 0.001). (**d**) The duration of active attack in pigs with GG (134.58 s), GT (247.15 s), and TT (255.51 s) genotypes of rs326221458 (H = 418.79, *p* < 0.001). Different letters between different genotypes indicate significant differences (*p* < 0.05).

There was a significant correlation (*p* < 0.05) in the aggressive behavior indicators between weaned pigs with different genotypes at rs326221458 on the JARID2 gene (Figure A1). The frequency and duration of standoff in pigs with the TT genotype of rs326221458 were greater than those with the GG or GT genotype (*p* < 0.05) (Figure 1a,b). The frequency of active attacks in pigs with the TT genotype of rs326221458 was significantly greater than that of pigs with the GG and GT genotypes (Figure 1c) (*p* < 0.05). The duration of active attacks in pigs with the TT or GT genotype of rs326221458 was significantly greater than that of the pigs with the GG genotype (Figure 1d) (*p* < 0.05). The 72 h arithmetic average of the four aggressive behavior indicators of pigs with TT genotype of rs326221458 was significantly higher than that of GG genotype pigs (Figure A1).

### 2.2. Expression Profile of Porcine JARID2 Gene and miR-9828-3p in Different Tissues

**Figure 2:**
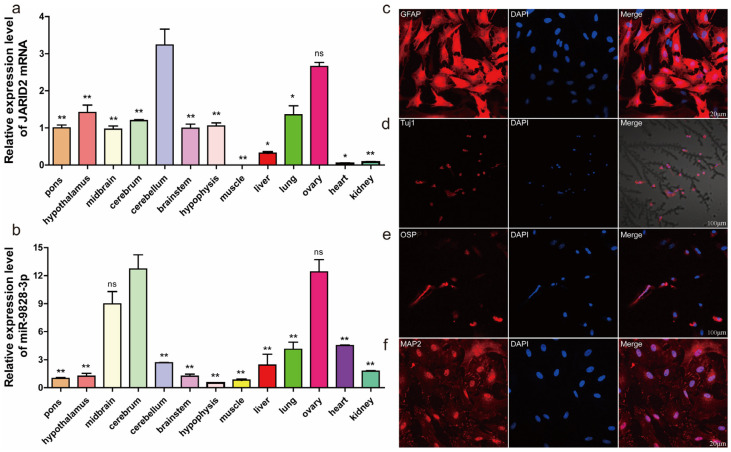
(**a**) The mRNA expression levels of the JARID2 gene in 13 tissues of 30-day-old pigs. (**b**) Expression levels of miR-9828-3p in 13 tissues of 30-day-old pigs. Data are represented as means ± SEM. Significance of the difference compared to the expression level in the cerebrum was indicated by a *p*-value of less than 0.05: * *p* < 0.05, ** *p* < 0.01, and ^ns^ *p* ≥ 0.05. (**c**) Immunofluorescence identification of PNCs. The PNCs were stained with GFAP (red) antibody, and nuclei were stained with DAPI (blue). (**d**) PNCs were immunostained with Tuj1 (red). (**e**) Immunostaining of PNCs with OSP (red). (**f**) PNCs were immunostained with MAP2 (red). The nuclei were stained with DAPI (blue).

The JARID2 gene exhibited the greatest expression level in the cerebellum, followed by the ovary, heart, and kidney, and the least in the muscle (Figure 2a). The expression level of miR-9828-3p was the greatest in the cerebrum, followed by the ovary, midbrain, liver, lungs, heart, kidneys, cerebellum, muscle, pons, hypothalamus, and brainstem, and the least in the hypophysis (Figure 2b).

### 2.3. Isolation, Culture, and Identification of Porcine Neural Cells

Porcine neural cells (PNCs) were isolated and cultured for 14 d, and identified by the immunostaining of glial fibrillary acidic protein (GFAP) and neuronal class III β-microtubule protein (Tuj1) (Figure 2c,d). The expression levels of outer surface protein (OSP) and microtubule-associated protein 2 (MAP2) were relatively low (Figure 2e,f). Based on the expression of specific proteins, the isolated and cultured PNCs were mainly composed of astrocytes, oligodendrocytes, microglia, and a small number of neurons.

### 2.4. MiR-9828-3p Targets the 3′-UTR of JARID2 mRNA

**Figure 3:**
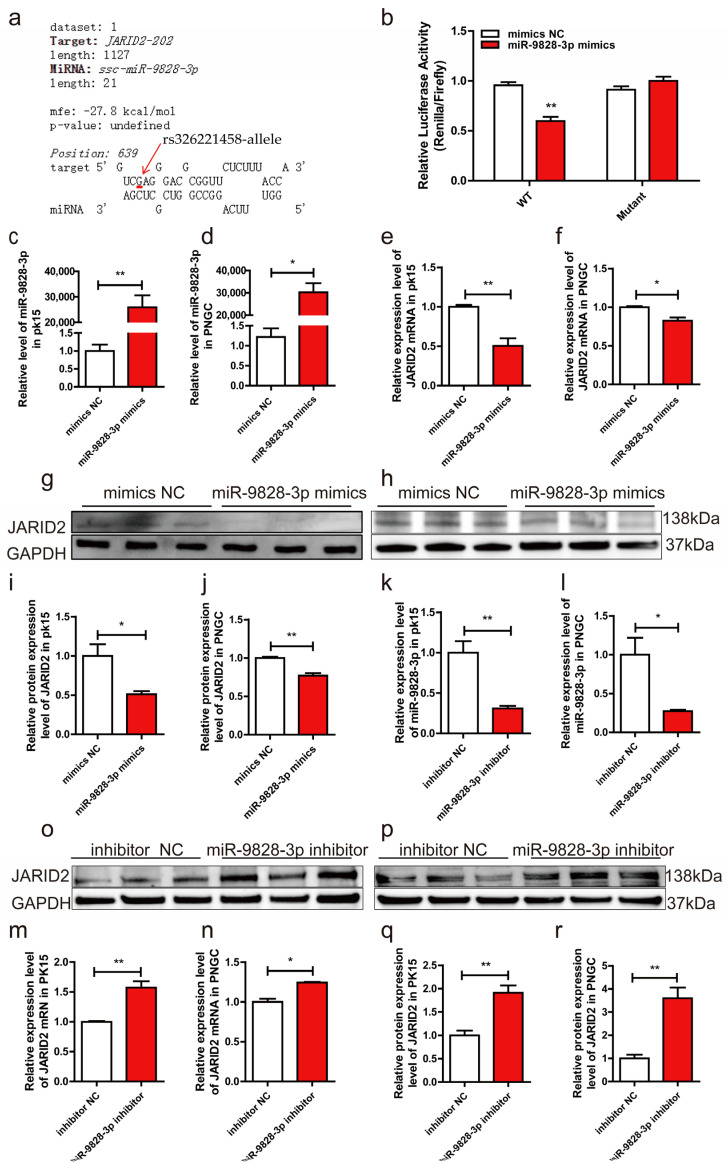
(**a**) RNAhybrid predictions indicated that miR-9828-3p binds to a sequence containing a G allele of rs326221458 with minimum free energy (MFE) binding of −27.8 kcal/mol. (**b**) Luciferase activities of the pmirGLO constructs carrying the porcine 3′-UTR with either the G or T allele of rs326221458 under co-transfection with miR-9828-3p mimics or mimics NC. Data are represented as means ± SEM, * *p* < 0.05, ** *p* < 0.01, three replicates per treatment. (**c**,**d**) PK15 cells and porcine neuroglial cells (PNGCs) were transfected with miR-9828-3p mimics or mimics NC, and the expression of miR-9828-3p mimics was quantified and normalized using U6 as the internal reference. (**e**,**f**) PK15 cells and PNGCs were transfected with miR-9828-3p mimics or mimics NC, and GAPDH was used as the internal reference. (**g**–**j**) PK15 cells and PNGCs were transfected with miR-9828-3p mimics or mimics NC, and GAPDH was used as the internal reference. (**k**,**l**) The expression levels of miR-9828-3p in PK15 cells and PNGCs transfected with miR-9828-3p inhibitor. (**m**,**n**) PK15 cells and PNGCs were transfected with either miR-9828-3p inhibitor or inhibitor NC. The mRNA level of the JARID2 gene was normalized using GAPDH as an internal reference. (**o**–**r**) PK15 cells and PNGCs were transfected with either miR-9828-3p inhibitor or inhibitor NC. The protein level of the JARID2 gene was normalized using GAPDH as an internal reference. Each treatment was repeated three times, and the results were expressed as the mean ± SEM. Significance was indicated by a *p*-value of less than 0.05, denoted by * *p* < 0.05 and ** *p* < 0.01.

**Figure 4:**
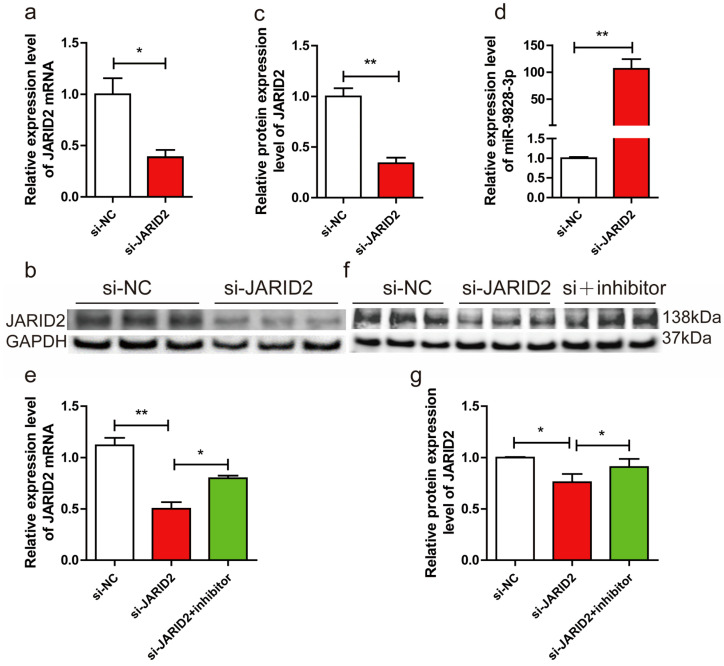
(**a**) The mRNA expression levels of the JARID2 gene, normalized using GAPDH as an internal reference, in porcine neuroglial cells (PNGCs) transfected with si-JARID2 or si-NC plasmid. (**b**,**c**) PNGCs were transfected with si-JARID2 or si-NC. The protein expression levels of the JARID2 gene were normalized using GAPDH as an internal reference. (**d**) PNGCs were transfected with si-JARID2 or si-NC. U6 was used as the internal reference to normalize the expression level of miR-9828-3p. (**e**–**g**) The mRNA and protein expression levels of the JARID2 gene, which were normalized using GAPDH as an internal reference, in PNGCs transfected with si-NC, si-JARID2, or si-JARID2 + miR-9828-3p inhibitor. All treatments were repeated in triplicate, and the results were expressed as the mean ± SEM. Significance was indicated by a *p*-value of less than 0.05, denoted by * *p* < 0.05 and ** *p* < 0.01.

RNAhybrid prediction indicated that miR-9828-3p binds to the 3′-UTR containing the G allele of rs326221458 with a minimum free energy (MFE) of −27.8 kcal/mol (Figure 3a). The WT and Mutant recombinant plasmids carrying the G and T alleles of rs326221458, respectively, were successfully constructed using the pmirGLO plasmid. In HEK-293T cells, the luciferase activity of the cells transfected with WT plasmid and miR-9828-3p mimics was significantly reduced compared with the other three treatments (Figure 3b). Results of qRT-PCR found that miR-9828-3p mimics were successfully transfected into PK15 cells and porcine neuroglial cells (PNGCs) (Figure 3c,d). Compared to the mimics NC groups, mRNA levels of the JARID2 gene were significantly down-regulated in PK15 cells and PNGCs (Figure 3e,f). Meanwhile, the protein expression level of the JARID2 gene was significantly down-regulated by miR-9828-3p (Figure 4g–j). qRT-PCR results indicated that the expression level of miR-9828-3p was significantly down-regulated by the miR-9828-3p inhibitor in both PK15 cells and PNGCs (Figure 3k,l). Compared with the inhibitor NC groups, the mRNA levels of the JARID2 gene were increased in the miR-9828-3p inhibitor groups in PK15 cells and PNGCs (Figure 3m,n). In addition, the protein expression levels of the JARID2 gene were also significantly up-regulated in the miR-9828-3p inhibitor groups compared to the inhibitor NC groups (Figure 3o–r).

### 2.5. Inhibition of miR-9828-3p Rescues Knocked-Down JARID2 Gene Expression

To test whether knocking down the JARID2 gene will affect the expression of miR-9828-3p, si-JARID2 or si-NC were transfected into PNGCs. Both mRNA and protein levels of the JARID2 gene were significantly down-regulated in the si-JARID2 group compared to the si-NC group (Figure 4a–c). A significant up-regulation of the expression level of miR-9828-3p was found in the si-JARID2 group compared with the si-NC group (Figure 4d). To test whether the expression level of the JARID2 gene could be rescued by miR-9828-3p inhibitor, si-NC, si-JARID2, and miR-9828-3p inhibitor+si-JARID2 were transfected into PNGCs, respectively. Surprisingly, both mRNA and protein expression levels of JARID2 were significantly up-regulated in the si-JARID2 + miR-9828-3p inhibitor group compared with the si-JARID2 group (Figure 4e,f).

### 2.6. MiR-9828-3p Inhibits Proliferation of Porcine Neuroglial Cells

**Figure 5:**
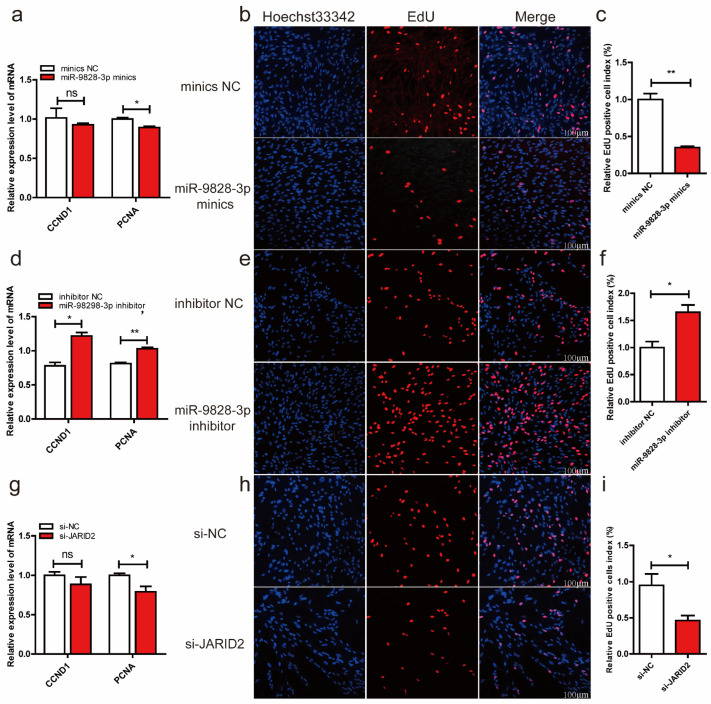
(**a**–**c**) Porcine neuroglial cells (PNGCs) were transfected with miR-9828-3p mimics or mimics NC. The mRNA expression levels of CCND1 and PCNA genes. (**d**–**f**) PNGCs were transfected with miR-9828-3p inhibitor or inhibitor NC. (**g**–**i**) PNGCs were transfected with si-JARID2 or si-NC. GAPDH was used as the internal reference to normalize the mRNA expression level of the CCND1 and PCNA genes. EdU (red) was used to measure the proliferation of cells, and nuclei were stained with Hoechst 33342 (blue). Scale bar = 100 μm, three replicates per treatment, all data are presented as mean ± SEM, * *p* < 0.05, ** *p* < 0.01, and ^ns^ *p* ≥ 0.05.

qRT-PCR results indicated that miR-9828-3p mimics significantly down-regulated the mRNA level of proliferating cell nuclear antigen (PCNA) in PNGCs (Figure 5a). The results of EdU showed that the relative EdU positive cell index was decreased in the miR-9828-3p mimics group compared with the mimics NC group (Figure 5b,c). Inhibition of miR-9828-3p significantly up-regulated the mRNA expression levels of cyclin D1 (CCND1) and proliferating cell nuclear antigen (PCNA) in PNGCs (Figure 5d). The relative EdU positive cell index was increased in the miR-9828-3p mimic group compared with the mimic NC group (Figure 5e,f). The mRNA level of the PCNA gene was down-regulated in PNGCs transfected with si-JARID2 plasmid (Figure 5g). Down-regulation of JARID2 by si-JARID2 inhibited the proliferation of PNGCs (Figure 5h,i).

## 3. Discussion

The human JARID2 gene is located in 6p22.3 and is associated with numerous psychiatric disorders and neurodevelopment. Deletion of the JARID2 haplotype leads to mental retardation and gait disorders [20]. Methylation of JARID2 is strongly associated with neurodevelopmental syndromes [21]. Previous research indicates a positive correlation between the number of short allelic repeat sequences on the JARID2 gene and SCZ. Aggressive behavior is more common in people with SCZ [22]. A comprehensive analysis of SNPs on chromosome 6 was conducted on 253 patients with bipolar disorder, 177 patients with major depressive disorder, and 119 patients with SCZ, as well as 986 healthy individuals. This study found that the JARID2 gene is a prominent susceptibility gene in SCZ patients [23]. In addition, a relationship between rs2235258 and rs9654600 on the JARID2 gene and the SCZ patients implied that JARID2 is an important psychosis gene in the population of the Changle area of Shandong Peninsula [24]. In addition to being related to SCZ, the JARID2 gene is also closely related to autism. Through analysis of 22,904 SNP sites on 2012 immune-related genes in humans, it was found that rs13193457 of the JARID2 gene is significantly related to autism [25]. These previous studies found that SNPs on the JARID2 gene are closely related to mental diseases. In the present study, SNP rs326221458, which is located in the 3′-UTR region of the porcine JARID2 gene, was closely associated with the aggressive behavior of weaned pigs. The frequency and duration of active attacks of weaned piglets with the TT mutant genotype of rs326221458 were significantly higher than those of piglets with the GG genotype. The duration of standoff and the frequency of being bullied among weaned pigs with the TT mutant genotype were significantly higher than those with the GG and GT genotypes. Since the duration of the standoff in pigs with TT and GT genotypes was almost equal, we suspect that the T gene has a dominant effect. Based on the above four behavioral indicators, we believe that the aggressive behavior of pigs with the TT mutant genotype is the strongest.

Porcine miR-9828-3p is located on chromosome 12 and encoded by 97 bases. Due to the high expression of miR-9828-3p in the cerebrum and midbrain, we hypothesized it involved physiological functions in the brain. The JARID2 gene has been reported to be highly expressed in the mouse cerebellum [26], which is similar to our findings. The cerebellum has the function of controlling the body’s balance, suggesting that the JARID2 gene may be involved in the regulation of the body’s balance. Since rs326221458 is located in the 3′-UTR region of the JARID2 gene, both SNPs and selective polyadenylation will affect the binding of miRNA and target mRNA [27]. Luciferase reporter results found that miR-9828-3p binds to the 3′-UTR region of the JARID2 gene, which proved that there is an interaction between the JARID2 gene and miR-9828-3p. Based on the dual-luciferase results, we hypothesize that pigs with the GG genotype exhibit lower RNA and protein expression levels of the JARID2 gene compared to those with the TT genotype. However, this requires confirmation through the identification of pigs with the corresponding genotype. Due to constraints, we have only demonstrated that the JARID2 gene’s protein expression is regulated by the rs326221458 locus via a dual-luciferase reporter assay. There are studies on SNPs affecting the binding of miRNA to target mRNA in psychiatric diseases and aggressive behavior. For example, the rs1321204 site on the serotonin receptor 1B (HTR1B) gene regulates the expression of HTR1B by affecting the binding of miR-96 to HTR1B, thus affecting the aggressive behavior of mice [28]. SNP rs895819, located on pre-miR-27a, is involved in the regulation of bipolar disorder by targeting neural cell adhesion molecule 1 (NCAM1) [29].

The JARID2 gene encodes a nuclear protein predominantly situated in the nucleus. However, it is also present in small quantities in the mitochondria and cytoplasm. The protein actively takes part in the regulation of various histone methylase complexes [30,31]. As a strong transcriptional repressor, JARID2 inhibits the proliferation of mouse pluripotent stem cells by down-regulating the mRNA of Murine Double Minute 2 (MDM 2) and Auxin Response Factors (Arf) [32]. Additionally, JARID2 also inhibits myogenic differentiation of rhabdomyosarcoma cells by binding to its upstream paired box 3- forkhead box (PAX 3-FOXO 1) protein [33]. During the development of glioma cells, the decrease of JARID2 leads to a reduction in phosphorylation levels of protein kinase B (Akt) and phosphatidylinositol-3-kinase (PI3K), which inhibits the proliferation, migration, and invasion of glioma cells [34]. JARID2, as a cancer-promoting gene, is highly expressed in various cancer cells. Down-regulation of JARID2 inhibits the proliferation of lung cancer cells, ovarian cancer cells, and prostate cancer cells [35,36,37]. In the present study, we found that miR-9828-3p inhibited the proliferation of PNGCs by down-regulating the expression of the JARID2 gene. Since neuroglial cells are involved in regulating synaptic plasticity, action potential conduction speed, information exchange efficiency, and other numerous nervous system activities [38], perhaps the proliferation of PNGCs is also a factor affecting the aggressive behavior of pigs. Recent research has demonstrated that astrocytes coordinate with neurons to regulate animal behavior by releasing gliotransmitters [39]. Astrocytes and microglia are known to be involved in the regulation of inflammation in the central nervous system and are also closely associated with Parkinson’s and Alzheimer’s diseases [40]. Astrocytes play irreplaceable roles in maintaining the blood-brain barrier, regulating synaptic activity, balancing neurotransmitters, and controlling neurotrophin secretion [41,42]. Previous studies found that bipolar patients have normal numbers and densities of oligodendrocytes but an increase in oligodendrocyte numbers compared to healthy controls [43]. In the present study, how the up-regulation of JARID2 gene expression alters pig neurons and thus affects pig aggression needs to be further investigated in vivo.

In conclusion, we found that the SNP rs326221458, located in the 3′-UTR region of the JARID2 gene, was associated with aggressive behavior in pigs. It was validated in porcine neuroglial cells, where this SNP regulated JARID2 expression by affecting the binding of miR-9828-3p to the mRNA of JARID2, but further validation in in vivo models is needed. Hence, the rs326221458 in the porcine JARID2 gene can be considered a new molecular marker for improving aggressive behavior in pigs.

## 4. Materials and Methods

### 4.1. Animals and Sample Collection

This study was approved by the Animal Use and Protection Committee of Nanjing Agricultural University (SYXK Su 2017-0027). A total of 500 weaned Suhuai pigs (268 barrows and 232 gilts) at 35 days of age with similar body weights were selected from the Huaiyin pig breeding farm. The Suhuai pig is a new hybrid breed that contains 75% European Large White boar blood and 25% Chinese native Huai sow blood. Before mixing, a camera with a memory card (Hangzhou Hikvision Digital Technology Co., Ltd., Hangzhou, China) was installed directly above the pig pen. At the time of group mixing, the 500 pigs were randomly divided into 51 pens, with 9–10 pigs in each pen, and numbers were marked on the backs of the pigs with a marker pen. Each pen was 2.5 m × 2.2 m in size and was equipped with a slit floor, stainless steel troughs, and nipple drinkers. The temperature, humidity, and light inside the pigsty were consistent with normal production. After 72 h of mixing, ear tissue samples were rapidly collected from experimental pigs with sterilized ear clippers, and the wounds were disinfected.

### 4.2. Behavioral Observation and Statistics

Behavioral observations and statistics were made for each pig based on a 72-h video of the pigs mixing. The pig behavioral indicators to be observed and counted are defined in advance, and all observers can master these behavioral indicators proficiently. Behavioral indexes mainly included frequency of standoff, duration of standoff(s), frequency of active attacks, and duration of active attacks(s). During a pig fight, an attack was considered one attack event if it lasted more than 3 s. After the fight exceeded 8 s, it was regarded as another new fighting event. The above-related aggressive behavior trait definitions are in (Table A1).

### 4.3. Single-Nucleotide Polymorphism Typing and Behavioral Association Analysis

In this study, a total of 182 pigs were randomly selected to verify potential functional SNP sites. Genomic DNA was extracted from the collected ear tissue samples using the DNA isolation mini kit. Primer5.0 was used to design specific primers (Table A2) for the 3′-UTR region of the porcine JARID2 gene. The DNA of the experimental porcine ear tissue samples was amplified by 1.1 × T3 Super PCR Mix (Qingke, Nanjing, China), and the amplification system consisted of 1 μL of Primer F, 1 μL of Primer R, 1 μL of DNA, and 22 μL of T3 Super PCR Mix. The amplification reaction program was as follows: 98 °C for 2 min, 98 °C for 10 s, 60 °C for 10 s, 72 °C for 10 s, and 72 °C for 2 min, a total of 35 cycles. The amplification products were sequenced by the Sanger method. The SNPs were analyzed and genotyped using the software DNAMAN 8.0 and Chromas 2.6. The SAS (https://welcome.oda.sas.com/login, accessed on 11 October 2021) online software was used to calculate the mean of the aggressive behavior indicators of pigs of different genotypes per hour after mixing. The least square mean of the generalized linear mixed model of the GLIMMIX program of SAS was used to detect the significance of aggressive behavior indicators in pigs of different genotypes.

### 4.4. Prediction of miRNAs in the 3′-UTR Region of the JARID2 Gene and Plasmid Construction

RNAhybrid (https://bibiserv.cebitec.uni-bielefeld.de/rnahybrid/, accessed on 11 October 2021) was used to predict the target miRNA in the 3′-UTR region of the JARID2 gene. Relevant experimental procedures were followed to construct the WT recombinant plasmid and mutant recombinant plasmid. The WT recombinant plasmid contains the rs326221458 G allele of porcine JARID2, and the mutant recombinant plasmid contains the rs326221458 T allele of porcine JARID2 gene. First, Primer3plus (https://www.primer3plus.com/, accessed on 11 March 2022) was used to design forward primers and reverse primers containing Sac I and Xba I recognition sites (Table A2). LAmp Master Mix (Vazyme Biotech, Nanjing, China) was used to amplify the porcine JARID2 gene DNA fragments of the G allele and T allele of the rs326221458 locus, respectively. The size of the amplified product is 202 bp. The amplified products were detected by agarose gel electrophoresis and sent for sequencing verification. Sequencing-correct amplification products were purified using the AXYGEN AxyPrepTM PCR Cleanup Kit (Vazyme Biotech, Nanjing, China). The purified PCR product was cloned into pmirGLO dual-luciferase miRNA target expression vector (Promega, Madison, Wisconsin, USA). The WT or mutant recombinant plasmid was transfected into DH5a competent cells for bacterial liquid PCR amplification and sent for sequencing verification. The correctly sequenced bacterial fluid was amplified, and the recombinant plasmid was extracted using the HiPure Plasmid EF Mini Kit (Magen, Guangzhou, China) and stored in the refrigerator at −20 °C. The recombinant plasmid was sequenced before transfecting cells to ensure whether the target fragment was inserted.

### 4.5. Cell Culture, Cell Transfection, and Luciferase Assays

After resuscitating HEK 293T (ATCC^®^ACS-4004™) cells, cells were inoculated with high sugar medium (DMEM/High, Gibco, Suzhou, China) containing 10% fetal bovine serum (FBS, Gibco, Suzhou, China) in culture flasks and cultured at 37 °C in a 5% CO_2_ incubator. When the cell density reached 60–70%, HEK293T cells were transfected with Opti-MEM (Gibco, Suzhou, China) dilution WT + miR-9828-3p mimics or mimics NC, Mutant + miR-9828-3p mimics or mimics NC (Gene-Pharma, Shanghai, China), (Table A3), and Lipofectamine 2000 (Invitrogen, Carlsbad, CA, USA).

The transfected cells were harvested 24 h after co-transfection, and HEK293T cells were lysed using passive lysis buffer. To verify the activity of miR-9828-3p towards SNP rs326221458, the luciferase activity of the lysates was measured using the Promega Dual-Luciferase System (Promega, Madison, Wisconsin, USA). Finally, the kidney luciferase activity of each sample was normalized to firefly luciferase, each treatment was repeated three times, and the results were expressed as mean ± SEM.

### 4.6. Culture of PK15 Cells, Isolation and Immunostaining Identification of Porcine Neural Cells

Porcine kidney-15 (PK15) cells were seeded in DMEM medium containing 10% FBS and cultured in a 37 °C, 5% CO_2_ incubator. When the cell density reached more than 90%, the cells were digested with trypsin and re-inoculated into 6-well or 12-well plates. When the cell density reached about 50%, small RNAs [miR-9828-3p mimics, mimics NC, miR-9828-3p inhibitor, inhibitor NC, si-JARID2, si-NC (Generay, Shanghai, China)] (Table A3) were transfected, and mRNA or protein were extracted at 30 h or 54 h after transfection.

A 30-day-old commercial pig was used to isolate and culture porcine neural cells (PNC). First, the pig was euthanized by covering its mouth and nose with ether-containing cotton, and the pig was rapidly decapitated. The entire brain was rapidly stripped with a scalpel and placed in a pre-cooled D-Hanks (Biosharp, Hefei, China) equilibration solution. After removing the blood and meninges from the brain, the brain tissue was cut into the cerebrum, cerebellum, hypothalamus, and mesencephalon using a scalpel and transferred to a test tube. The same volume of 2 mg/mL papain (Biosharp, Hefei, China) as that of the brain tissue samples was added and cut into 1 mm^3^ pieces. Then 2 mg/mL papain and DNAse I (Sigma Aldrich, St. Louis, MO, USA) (10 μg/mL) were mixed in a ratio of 6:1, added to the chopped tissues, and digested at 37 °C for 30 min. The digestion was terminated with F12 (Biosharp, Hefei, China) medium containing 10% FBS in the same volume as the digested solution, iltered through a 40 μm cell sieve, washed with PBS, and centrifuged. Finally, the suspension of F12 medium containing 15% FBS was inoculated into T25 culture flasks and incubated in a 5% CO_2_ incubator at 37 °C. After inoculation of PNCs for 36 or 24 h, the original medium was replaced with fresh medium.

When the density of PNCs seeded on the cell slide reached about 80%, the cells were fixed with 4% paraformaldehyde at room temperature for 30 min. Then the cells were permeabilized with 0.3% TritonX-100 at room temperature for 20 min and blocked with Immunol Staining Blocking Buffer for 1 h. Anti-Tuj1 (neuronal class III β-microtubule protein) antibody, anti-MAP2 (Microtubule-associated protein 2) antibody (Beyotime, Shanghai, China), anti-OSP antibody, and anti-GFAP antibody (1:100, Abclonal, Wuhan, China) were diluted with immunofluorescent antibody diluent (Beyotime, Shanghai, China). Immunostaining of PNCs was carried out with the four diluted antibodies. The PNCs were incubated in the dark with a secondary antibody (goat-anti-rabbit antibody, 1:500, Biosharp, Hefei, China) for 1 h. Cell nuclei were stained with DAPI for 20 min under light-avoiding conditions. Then, 4 μL of fluorescence quenching mounting agent was added dropwise on a slide, the side containing the cells attached to the fluorescence quencher, and the staining under confocal light was observed.

### 4.7. Total RNA Extraction, Inversion, and qRT-PCR

According to the manufacturer’s instructions, TsingZol RNA Reagent (Qingke, Nanjing, China) was used to extract total RNA from porcine tissue samples or cells (PK15 and porcine neuroglial cells). The purity and concentration of the extracted total RNA were determined using a NanoPhotometer^®^ Spectrophotometer (IMPLEN, Westlake Village, CA, USA). Total RNA was reverse transcribed into cDNA using the HiScriptIIQ RT SuperMix (Vazyme Biotech, Nanjing, China). MiRNA was reverse transcribed into cDNA using the miRNA 1st Strand cDNA Synthesis Kit (Vazyme Biotech, Nanjing, China) at temperatures of 42 °C for 2 min, 25 °C for 5 min, 55 °C for 15 min, and 85 °C for 5 min, respectively. Amplification reactions were performed with cDNA as a template according to the SYBR Green Master Mix (Vazyme Biotech, Nanjing, China) reaction system at 95 °C for 5 min, 95 °C for 10 s, and 60 °C for 30 s. The relative mRNA expression was calculated by 2^−ΔΔCt^. Each treatment was repeated three times. The expression levels of coding genes were normalized by GAPDH or U6. The amplification primers used are detailed in the attached table (Table A2).

### 4.8. Western Blotting

The cell culture medium was discarded and washed with PBS. RAPI lysis buffer containing PMSF was added, and the cells were lysed on ice for 30 min. After centrifugation at 12,000 rpm for 10 min, the protein concentration was determined by absorbance at 562 nm using BCA (Biosharp, Hefei, China). Then 5 × SEMS-PAGE buffer (5:1) was added, and the samples were denatured at 96 °C for 15 min. Protein extracts were separated using 8% sodium dodecyl sulfate polyacrylamide gel electrophoresis (SDS-PAGE) gels at corresponding voltages and currents (Genscript, Biotechnology, Piscataway, NJ, USA). The protein-containing gel was transferred to methanol-activated polyvinylidene fluoride (PVDF, Millipore, Burlington, Massachusetts, USA) membranes, blocked with Rapid Blocking Solution (Beyotime, Shanghai, China), and incubated overnight with JARID2 (1:1000, Abclonal, Wuhan, China) antibody or GAPDH (1:4000, Affinity, Nanjing, China) antibody. The secondary antibody was then added and incubated for 1 h at room temperature. ECL substrate A and peroxide solution B (1:1, Vazyme Biotech, Nanjing, China) were used for color development in the Image LAS-4000 system. Quantify protein bands using software ImageJ1.8.0.112.

### 4.9. EdU Detects the Proliferation of Porcine Neuroglial Cells

Porcine neuroglial cells (PNGCs) are seeded in 12-well plates containing cell sheets. Transfection was started when the cell density reached 60%. After 30 h of transfection, EdU (Cy5) was diluted with 10% FBS in F12 medium (APExBIO, Houston, TX, USA) to a final concentration of 10 μM EdU in the medium and incubated in an incubator for 4 h. The fixative was removed, and the cells were washed twice with 3% BSA for 5 min each time. Soak the PNGCs in 0.3% Triton^®^X-100 for 20 min at room temperature. Configure the desired Click reaction solution with 860 μL of 1X EdU Reaction Buffer, 40 μL of CuSO_4_, 1 μL of Cy5 azide, and 100 μL of 1 × EdU Buffer Additive per ml. Click reaction solution was added to each well and incubated in the dark for 30 min at room temperature. Incubation was completed with Hoechst 33342 at a final concentration of 5 μL/mg for 25 min at room temperature in the dark. After staining, Cy5 azide and Hoechst 33342 were photographed under a confocal microscope using excitation wavelengths of 646 nm and 350 nm, respectively.

### 4.10. Statistical Analysis

Indicators of aggressive behavior of pigs with different genotypes of the linked SNPs were determined using the GLIMMIX procedure with the model option DIST = EXPO in SAS 9.04.01M7P08062020 online software (https://welcome.oda.sas.com/login, accessed on 11 October 2021) with the sex, parity, genotype, and initial body weight as fixed effects. A nonparametric test (Kruskal–Wallis test) was used for the comparison of aggressive behavior between pigs with different genotypes in Figure A1. The significance of relevant cell experiments was analyzed using an unpaired, two-sided student’s *t*-test. The results were expressed as the mean ± SEM, and a *p*-value of less than 0.05 indicated a statistically significant difference.

## Data Availability

The datasets generated and/or analyzed during the current study are available from the corresponding author upon reasonable request.

## Appendix A

**Table A1:** Ethogram used to evaluate the aggressiveness of pigs after mixing.

Behavior	Description
Fight	A vigorous biting, head-knocking, or displacement with the physical contact of two individuals for more than 3 s and intervening periods of less than 8 s; otherwise, the fight was interrupted. In a fight, a pig shows active attack, then the other pig shows being bullied, or both show standoff behavior.
Active attack	In a fight, a pig actively gives a biting, pushing, or chasing to another pig.
Standoff	In a fight, if the two pigs stand side by side, shoulder to shoulder, and one pig throws his head to the head or neck of the other pig, there is an aggressive interaction with no dominance sign produced by either pair member at any time.

**Table A2:** Primers of plasmid construction and RT-qPCR for porcine JARID2 gene.

Primer	Primer Sequence (5′-3′)	Product Size (bp)	Start	Stop	Usage
JARID2-3′UTR-P1	F:CGGGCGAGGGATGGATATTTA	565	15	35	3′UTR-PCR
	R:AAACACAACGGGGAGTCTCC		579	599	
JARID2-3′UTR-P2	F:TTTCTTTGGGCGGTG ATGGC	503	577	596	3′UTR-PCR
	R:ATAGACCAGATGAGCGCAGA		1080	1100	
JARID2 WT and MUT	F:CGAGCTCGGTTGAAACCGGAGACTCCCC	202			miR-9828-3p vector construction
	R:GCTCGATAGCCACAGGTTTCCCTCACGTGA				
JARID2	F:GCTCAATCCCAGCCGAATAG	338			RT-qPCR
	R:GCTGGAACCATTGAAAACAT				
GAPDH	F:CAAGGAGTAAGAGCCCCTGG	181			RT-qPCR
	F:GGTACATGACGAGGCAGGTC				
miR-9828-3p	F: GGUUUCAGGCCGGUCGCUCGA				RT-qPCR
	R:CAGCCACAAAAGAGCACAAT				
U6	F:GCTTCGGCAGCACATATAC	86			RT-qPCR
	R:TTCACGAATTTGCGTGTCAT				
PCNA	F:TGCAGATGTACCCCTTGTTGT	164			RT-qPCR
	R:TATGTGCTGGCATCACCGAA				
CCND1	F:ATCAGGTGTGACCCGGACT	184			RT-qPCR
	R:CGCCTCAAATGTTCACGTCG				

**Table A3:** The information of oligonucleotides.

Sequence Name	Sense (5′-3′)	Antisense (5′-3′)
Si-JARID2-2	GCGGCAAAGUGGACACCAACA	UUGGUGUCCACUUUGCCGCAG
Si-NC	UUCUCCGAACGUGUCACGUTT	ACGUGACACGUUCGGAGAATT
miR-9828-3p mimics	GGUUUCAGGCCGGUCGCUCGA	TCGAGCGACCGGCCTGAAACC
mimics NC	UUCUCCGAACGUGUCACGUTT	ACGUGACACGUUCGGAGAATT
miR-9828-3p inhibitor	UCGAGCGACCGGCCUGAAACC	GGTTTACAGGCCGGTCGCTCGA
inhibitor NC	CAGUACUUUUGUGUAGUACAA	TTGTACTACACAAAAGTACTG
